# Trends of colorectal cancer surgery in 2022

**DOI:** 10.1093/bjsopen/zrad005

**Published:** 2023-02-13

2022 was a great year for high-quality submissions to *BJS Open* in the field of colorectal cancer (CRC) surgery. Layfield and associates^1^ documented how changes in treatments delivered by the multidisciplinary team (MDT) over 14 years, demonstrating how implementation of CRC management based on the latest gold standards, significantly improved survival and reduced mortality in a high-volume UK institution. Of note, concerning older patients, they demonstrated the changing pattern in MDT decisions with a decreased rate of surgical intervention. Despite this reduction in surgery and oncological therapies, the survival benefit was also seen among patients aged 80 years or more, which may reflect the ‘*inclusion of patients who would previously have undergone surgery but lived a little longer without it*’ or had fatal complications after major procedures.

Regarding management, in their analysis of 41 800 patients from Denmark and Yorkshire (UK), Taylor and co-authors^2^ reported the application of specific policies in Denmark at a national level that resulted in a reduction in left-sided emergency resections and an increase in stenting. This resulted in conversion of potential emergency into elective resections and reduced 30-day postoperative mortality.

The debate on management leads to other important topics: cost-effectiveness and correction of modifiable preoperative risk factors with protocols implemented at institutional level.

In particular, a large Canadian study^3^, analysing circular stapler anastomotic rings specimens from nearly 490 CRC resections, confirmed that their routine pathologic evaluation is not useful, as no patients had cancer in the ring specimen, 5.1 per cent had benign pathological findings and patients’ management was never affected by this result. Also, in another registry study including nearly 6200 patients from Sweden^4^, authors documented that the routine use of rectal washout during anterior resection did not impact the 3-year oncological outcomes, even if a reduction in local recurrence risk after the 5-year follow-up was observed.

In another large Danish study^5^, authors investigated the effect of screening for modifiable high-risk factors combined with targeted interventions in CRC surgery. These consisted of a screening for anaemia, low functional capacity, and nutritional status and their implementation (iron supplementation, pre-habilitation, nutritional supplements, and consultation with a dietician) for a minimum of 4 weeks before surgery. Even when analyses were balanced for age, sex, smoking habits, stage of disease, ASA score, surgical approach, and surgical procedure, the intervention was associated with a 10.9 per cent absolute risk reduction of a complicated postoperative course, primarily due to a reduction in severe complications, highlighting what and how it's worth to correct.

Then, if not preventable, complications could be diagnosed early, and anastomotic leak is the Achilles’ heel of every CRC procedure. In this field, *BJS Open* recently published two beautiful basic science papers: one Chinese focused on six inflammatory factors (including interleukin (IL)-1β, IL-6, IL-10, tumour necrosis factor (TNF)-α, matrix metalloproteinase (MMP)2, and MMP9) in the peritoneal drainage fluid^6^, and another from Sweden^7^ providing a proteomic analysis on serum preoperative biomarkers of inflammation in relation to anastomotic leak. Although data need to be confirmed and externally validated, both studies provided new insight on this fearsome complication.

Finally, we should not forget that the goal of CRC treatment should be first and foremost patient-centred. In this regard, a large cross-sectional study^8^ interviewing more than 2500 patients from ten countries, reported how patients perceive life with a colostomy. Authors documented several differences among countries and that one-quarter of the patients had an impaired quality of life, affecting in particular those reporting stoma dysfunction, financial burden from the stoma, unemployment, being single/widowed and young age.
